# Catechin in Human Health and Disease

**DOI:** 10.3390/molecules24030528

**Published:** 2019-02-01

**Authors:** Mamoru Isemura

**Affiliations:** Tea Science Center, University of Shizuoka, Suruga-ku, Shizuoka 422-8526, Japan; isemura@u-shizuoka-ken.ac.jp

Catechin, the name of which is derived from catechu of the extract of *Acacia catechu* L., is 3,3’,4’,5,7-pentahydroxyflavan with two steric forms of (+)-catechin (Figure 1) and its enantiomer [1,2]. In addition, in a broad sense, catechin represents the chemical family name of the compounds derived from catechin. Catechins are distributed in a variety of foods and herbs including tea, apples, persimmons, cacaos, grapes, and berries. This special issue is devoted to information on catechin’s activities related to human health.

Tea, a product obtained from the leaves and buds of the plant *Camellia sinensis*, is one of the richest catechin sources and contains, as the major catechin, (−)-epigallocatechin-3-gallate (EGCG) (Figure 1) which has many beneficial properties for human health such as anticancer, anti-obesity, antidiabetic, anticardiovascular, anti-infectious, hepatoprotective, and neuroprotective effects. A number of human epidemiological and clinical studies on tea have provided evidence for its anticancer benefits and these results have been supported by cell-based and animal experiments, although studies that show conflicting results have also been reported. In addition, detailed molecular mechanisms have been proposed for the action mechanism of EGCG and other catechins. One of the most attractive mechanisms is the one in which reactive oxygen species (ROS) is involved. EGCG is known to have dual actions in relation to ROS as an anti-oxidant and a pro-oxidant. Several lines of evidence have indicated that EGCG can both eliminate ROS by scavenging and enhance ROS production. 

In this special issue, Bernatoniene and Kopustinskiene [2] reviewed the biochemical properties of catechins, their anti-oxidant activity, and the mechanisms of action involved in the prevention of oxidative stress-caused diseases such as cancer, cardiovascular diseases, and neurodegenerative diseases.

With respect to the anticancer effect of green tea catechins, Shirakami and Shimizu [3] provided updated information on diverse mechanisms, including anti-oxidative, pro-oxidative, and anti-inflammatory activities, immune and epigenetic modification, and receptor tyrosine kinase inhibition. They pointed out that it is unclear whether the in vitro observation with high concentrations of EGCG can be directly extrapolated to cancer chemoprevention in animals and humans because of its low bioavailability.

Among neurodegenerative diseases, Alzheimer’s disease (AD) is one of the most common disorders worldwide. Oxidative stress is a component of the pathological mechanism underlying AD. It can be caused by a disruption of the balance between ROS and anti-oxidant molecules. This imbalance may also cause neuroinflammation. Ide et al. [4] summarized updated information and perspectives of the effects of catechins on AD based on molecular mechanisms, including those related to the anti-oxidative, anti-inflammatory, protein kinase C-related, and neurotransmission-related properties of catechins. 

Similarly, Pervin et al. [5] summarized recent findings on the beneficial effects of catechins on neurodegenerative diseases. Although several human studies have supported these effects, others have not. These authors suggest that the discrepancy may be due to the incomplete adjustment of confounding factors, including the method of quantifying consumption, beverage temperature, cigarette smoking, alcohol consumption, and differences in genetic and environmental factors, such as race, sex, age, and lifestyle. This issue may be applied to human epidemiological studies on other diseases including cancer. 

Several epidemiological studies have suggested that the regular consumption of green tea decreases influenza infection rates and some cold symptoms, and that gargling with tea catechins may protect against the development of influenza infection. Furushima et al. [6] reviewed the effect of tea catechins on influenza infection and the common cold by focusing on epidemiological/clinical studies, and indicated the need for further studies to confirm clinical efficacy. 

In regard to anti-obesity activity, many studies have shown that EGCG in green tea, methylated EGCG in oolong tea, theaflavins in black tea, and polyphenol metabolites in dark tea exhibit weight-loss properties. Rothenberg et al. [7] proposed a “Short Chain Fatty Acid (SCFA) hypothesis” to explain how various tea types can all effectively induce weight loss. SCFAs generated in the gut through reactions among undigested carbohydrates, catechins, and gut microbiota may enhance lipid metabolism through AMP-activated protein kinase activation, leading to their anti-obesity activity.

To gain effective concentrations of EGCG and other catechins, studies are important to reveal the efficacy of chemical modification, delivery systems, and synergy with other agents. Previous papers have shown that chemical modification is one of the promising methods to enhance their biological effect as exemplified by peracetylated EGCG which potently suppresses colon tumorigenesis in mice [8]. Kaihatsu et al. [9] summarized the antiviral activity of EGCG and proposed a newly developed EGCG-fatty acid derivative as an efficient agent. EGCG-fatty acid monoesters showed improved antiviral activities against different types of viruses, probably due to their increased affinity for virus and cellular membranes.

Mukherjee et al. [10] showed that a mixture of curcumin, EGCG, and resveratrol in a liposomal form is a potential onco-immunotherapeutic agent against glioblastoma, although curcumin alone has limited antitumor efficacy in vivo due to its low bioavailability like EGCG. 

Shi et al. [11] provided an overview of materials and techniques used in encapsulating EGCG. The stability, bioavailability, and function of EGCG can be improved by encapsulation. EGCG encapsulated in proteins showed a sustained release partly due to the inhibition of the activity of digestive enzymes. EGCG encapsulated in carbohydrates showed improved mucoadhesion, intestinal permeation, tissue-targeting delivery, and inhibition of active efflux. EGCG encapsulated in lipids showed improved stability and sustained release, and was directly taken up by epithelial cells. Thus, encapsulation of EGCG with food grade materials would be useful to improve the bioavailability and functionality of EGCG.

An excessive dose of catechins may cause unfavorable effects such as hepatitis [12]. Kaleri et al. [13] reported that dietary copper can reduce the hepatotoxicity of EGCG, possibly by up-regulating ceruloplasmin activity leading to reduced levels of ROS, suggesting its usefulness in promoting EGCG applications.

The chemical modification of gelatin using EGCG promotes bone formation in vivo. Honda et al. [14] proposed that fabricated EGCG-modified gelatin sponges (EGCG-GS) have the potential to be applicable to regenerative therapy. Their study suggested that vacuum heating enhances the bone forming capability of EGCG-GS, possibly through the dehydrothermal cross-linking of EGCG-GS, which provides a scaffold for cells, leading to the sustained pharmacological effect of EGCG. 

In addition to ROS-related mechanisms, catechin–protein interaction is believed to be involved in the mechanisms by which catechins exert their biological activities. Saeki et al. [15] reviewed how EGCG–protein interactions can explain the mechanism by which green tea/EGCG can exhibit health beneficial effects. Several methods including dot assays, affinity gel chromatography, surface plasmon resonance, computational docking analyses (CDA), and X-ray crystallographic analysis (XCA) have provided evidence to show EGCG–protein interactions and how EGCG can fit or occupy the position in or near functional sites and induce a conformational change, including a quaternary conformational change. These authors suggest EGCG as a lead compound for drug design. 

Nakano et al. [16] discussed how CDA and XCA, among the above-mentioned methods, are useful in new drug design strategies of catechins. CDA and XCA have revealed that the galloyl moiety anchors catechin to the cleft of proteins through interactions with its hydroxyl groups, explaining the higher activity of galloylated catechins such as EGCG and epicatechin gallate as compared to non-galloylated catechins (Figure 1). 

Shimamura et al. [17] revealed the interaction between catechins and *Staphylococcal* enterotoxin A using surface plasmon resonance, Fourier transform infrared spectroscopy, isothermal titration calorimetry, and CDA. The data indicated that the hydroxyl group at position 3 of the galloyl group in the catechin structure is responsible for binding affinity with the Tyr91 in the toxin’s active sites, providing valuable information related to the prevention of food-derived poisoning.

Cross-sectional and retrospective evidence indicates that tea consumption can mitigate bone loss and reduce the risk of osteoporotic fractures. Chen et al. [18] proposed that EGCG might be an important nutrient in modulating bone resorption, since EGCG at 1–10 μM decreased osteoclastogenesis and tartrate-resistant acid phosphatase activity through the receptor activator of the nuclear factor-kB (RANK)/RANK ligand/osteoprotegrin pathway. Lin et al. [19] reported that EGCG can increase mRNA expression of bone morphogenetic protein 2 and subsequent osteogenesis-related genes including alkaline phosphatase, osteonectin, and osteocalcin, leading to enhanced mineralization.

Chen et al. [20] examined the stability and bioaccessibility of polyphenols from the bark of *Acacia mearnsii* after in vitro treatments mimetic to gastrointestinal digestion. After simulated intestinal digestion, the total polyphenol content and biological activities decreased significantly compared to those of the non-treated extract, which was attributed to the degradation of proanthocyanidins. Nevertheless, retention of the anti-oxidant capacity and α-glucosidase-inhibitory activity suggests that polyphenols derived from the plant appear to have the potential to be useful for human health. 

“Kangzhuan” is the most popular type among Tibetan tea products. Xie et al. [21] reported that a lyophilized aqueous extract of “Kangzhuan” possesses anti-oxidative or cytoprotective properties. These effects may be attributed mainly to the presence of phenolic components, including gallic acid and four catechins. These phenolic components may undergo electron transfer, H^+^-transfer, and Fe^2+^-chelating pathways to exhibit anti-oxidative or cytoprotective effects.

Theaflavin and its galloyl esters are the red pigments in black tea that possess a variety of health benefits similar to those found in green tea catechins. Several biosynthetic methods have been developed for the mass production of theaflavins. Takemoto and Takemoto [22] provided updated information on synthetic methods for theaflavins and their health benefits, encouraging future studies to reveal their detailed action mechanism and to develop new supplements. 

## Figures and Tables

**Figure 1:**
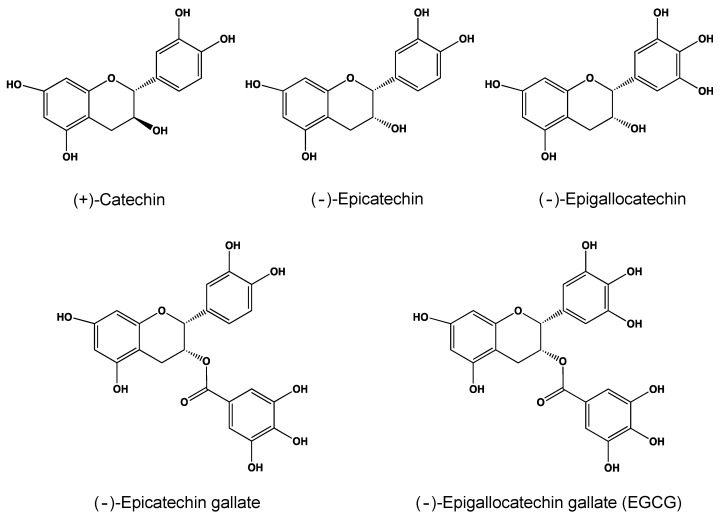
(+)-Catechin and major green tea catechins.
